# Electrophysiological Characteristics of the LQT2 Syndrome Mutation *KCNH2-G572S* and Regulation by Accessory Protein *KCNE2*

**DOI:** 10.3389/fphys.2016.00650

**Published:** 2016-12-27

**Authors:** Li Liu, Jinwen Tian, Caiyi Lu, Xi Chen, Yicheng Fu, Bin Xu, Chao Zhu, Yanmei Sun, Yu Zhang, Ying Zhao, Yang Li

**Affiliations:** ^1^Department of Cardiology, General Hospital of People's Liberation ArmyBeijing, China; ^2^The Third Department of Internal Medicine, Beijing Municipal Corps Hospital of Chinese People's Armed Police ForceBeijing, China

**Keywords:** long QT syndrome type 2, *KCNH2*, *KCNE2*, action potential, low temperature, trafficking

## Abstract

Mutations in *hERG* cause long QT syndrome type 2 which is characterized by a prolonged QT interval on electrocardiogram and predisposition to life-threatening ventricular tachyarrhythmia, syncope, and sudden death. *hERG-G572S* induces trafficking defects of *hERG* channel protein from Golgi to the plasma membrane and results in a dominant negative suppression of *hERG* current density. As an accessory β subunit, *KCNE2* promotes *hERG* migration from Golgi to cellular membrane. In this study, we investigated the rescue effect of *KCNE2* in a *G572S* mutation of *hERG*. Transfection was performed into HEK293 cells. Patch clamp technique, western blotting analyses and confocal microscopic examination were used. Results showed that *KCNE2* had a significantly enhanced effect on *G572S* mutation current. The increase of current was largest at *KCNH2*:*KCNE2* of 1:3. Confocal images showed co-expressing *G572S* and *KCNE2* could cause a substantial up-regulated membrane protein (155 kDa) expression. Expression of membrane protein accumulated markedly with increasing ratio of *KCNH2*:*KCNE2*. *G572S* defective mutant could be restored by both *KCNE2* and lower temperature (27°C), which suggested that the lower temperature could be the favorable circumstances for the rescue function of *KCNE2*. In this study, we successfully set up “the action potential” on the HEK 293 cells by genetically engineered to express Kir2.1, Nav1.5, and Kv11.1, wherein on reaching over an excitation threshold by current injection. The results suggested that *KCNE2* could shorten action potential duration which was prolonged by *G572S*. These findings described electrophysiological characteristics of the LQT2 syndrome mutation *KCNH2-G572S* and regulation by accessory protein *KCNE2*, and provided a clue about LQT2 and relative rescue mechanism.

## Introduction

Human hereditary long QT syndrome (LQTS) is a heterogeneous cardiac repolarization disorder characterized by a prolonged QT interval on the surface electrocardiogram (Schwartz et al., 2012). The human-ether-a-go-go-related gene (*hERG* or *KCNH2*) encodes the pore-forming alpha subunit of K^+^ channel that resembles the rapid component of delayed rectifier current (I_Kr_) in cardiac myocytes (Warmke and Ganetzky, 1994; Sanguinetti et al., 1995; Trudeau et al., 1995). Mutations in *hERG* cause long QT syndrome type 2 (LQT2) (Sanguinetti et al., 1995; Schweigmann et al., 2014), which is characterized by a prolonged QT interval on electrocardiogram and predisposition to life-threatening ventricular tachyarrhythmia, syncope and sudden death (Saenen and Vrints, 2008).

In 1999, Abbott et al. described that MiRP1 formed stable assemblies with *hERG* and the resulting channel complex had functional attributes like those of native, cardiac I_Kr_ channels. There are discrepancies among investigators in describing the effects of *KCNE2* on *hERG* currents (Abbott et al., 1999; Mazhari et al., 2001; Weerapura et al., 2002; Eldstrom and Fedida, 2011). Previous studies showed that *KCNE2* could modulate single channel conductance (Abbott et al., 1999), gating kinetics (Zhang et al., 2001), the rate of protein degradation (Zhang et al., 2012), regulate *hERG* endocytosis, alter some aspects of *hERG* pharmacology (Dupuis et al., 2005) and promote *hERG* migration from Golgi to cellular membrane as an accessory β subunit (Um and McDonald, 2007).

It is estimated that at least 80% of missense mutations in *KCNH2* result in defects in assembly and hence trafficking of functional channels to the plasma membrane (Anderson et al., 2006; Walker et al., 2007). The considerable work has been undertaken looking at pathways of *hERG* trafficking and the role of chaperones in detecting misfolded mutant proteins (Thomas, 2003). To our knowledge, *hERG-G572S* induces trafficking defects of *hERG* channel protein from Golgi to the plasma membrane and results in a dominant negative suppression of *hERG* current density. *hERG-G572S* channel subunits can coassemble with *WT* subunits, and this results in retention of *WT* subunits within the ER. *In silico* modeling it is found that *G572S* would cause a significant prolongation of AP duration (Zhao et al., 2009).

As mentioned above, we speculated *KCNE2* could have some effects on *hERG-G572S*. This study sought to research electrophysiological characteristics of *KCNH2-G572S* and regulation of *KCNE2* on *KCNH2-G572S* mutation.

## Materials and methods

### Site-directed mutagenesis and transfection into HEK293 cells

The *G572S* mutation was introduced into the *KCNH2* cDNA by directed mutagenesis (QuickChange Site-Directed Mutagenesis kit, Stratagene). The Wild-type (*WT*) and mutant *SCN5A* were also inserted in frame into a pcDNA3.1 vector plasmid (Invitrogen) to be expressed. Sequence analysis was used to confirm the presence of the mutation. *WT* and mutant of *KCNH2* were transiently transfected in Human embryonic kidney (HEK)-293 cells. HEK293 cells were transiently transfected with 1.2 μg of the *KCNH2* construct, either *WT* or *G572S*, using Lipofectamin 2000 (Life Technologies, Gaithersburg, MD, USA) according to the manufacturer's protocol. The green fluorescent protein (*GFP*) gene was as a reporter gene in co-transfection HEK293 cells with plasmid. After 6 h, the transfection medium was replaced with regular medium. Transfected HEK293 cells were cultured for 48–72 h. Only cells exhibiting green fluorescence were separated by enzymatic treatment and seeded in plastic Petri dishes bottomed with a coverslip. Using the patch-clamp technique, currents were recorded.

### Patch clamp recording

*hERG* currents were recorded at room temperature using the whole-cell patch-clamp configuration with the MultiClamp 700B amplifier (Axon Instruments). Data were sampled at 10 kHz and filtered at 5 kHz subsequently for analysis (Digidata 1440A, Axon Instruments). Recording pipettes were pulled from borosilicate capillary tubes by using P-97 programmable patch micropipette horizontal puller (Sutter Instruments). Micropipette resistance ranged from 2.0 to 5.5 MΩ when filled with the internal solution and immersed in the external solution. To minimize voltage clamp errors, series resistance (Rseries) was compensated by <2.0 MΩ and usually ≥80% compensation was achieved in all experiments. The membrane capacitance was compensated by approximately 80~90% of their initial value. The internal solution contained (in mM): K aspartate 85, KCl 45, Na pyruvate 5, K_2_ATP 3, MgCl_2_ 4, EGTA 10, HEPES 10, and glucose 11, adjusted to pH 7.2 with KOH. Cells were perfused with an external (bath) solution containing (in mM): NaCl 140, CaCl_2_ 1, MgCl_2_ 1, KCl 4, HEPES 10, and glucose 5, adjusted to pH 7.4 with NaOH.

To record the I_Na_, cells were bathed in a solution that contained (in mM) NaCl 40, CaCl_2_ 2, CsCl 5, MgCl_2_ 1.2, HEPES 10, and glucose 5, adjusted to pH 7.4 with CsOH. The glass pipettes were filled with a solution of (in mM) CsCl 60, caesium aspartate 80, EGTA 11, HEPES 10, Na_2_ATP 5, at pH 7.2, adjusted with CsOH. To avoid current drift of I_Na_, sodium ion concentration of external (bath) solution was set at 40 uM, room temperature was kept at 20°C, micropipette of 0.5 μm-inner diameter was used, and micropipette resistance ranged from 0.5 to 1 MΩ when filled with the internal solution and immersed in the external solution (Sokolov et al., 2013).

To record AP, cells were bathed in a solution that contained (in mM) NaCl 137, KCl 5.9, CaCl_2_ 2.2, MgCl_2_ 1.2, glucose 14, and HEPES 10. The pH of the solution was adjusted to 7.4 with NaOH. The pipette solution for whole-cell recordings contained (in mM) KCl 140, MgCl_2_ 4, HEPES 10, Na_2_ATP 2, and EGTA 0.05. The pH of the pipette solution was adjusted to 7.2 with KOH (Fujii et al., 2013).

### Western blotting analyses

Total homogenates were obtained from HEK293 cells using standard methods (Zhou et al., 1998). Blots were probed with primary antibodies against Kv11.1. Chemiluminescent detection was performed with substrate reagents from Pierce Biotechnology. Densitometric analysis was performed with Image for Windows software (V. Beta 4.0.2; Scion). The expression level of Kv11.1 protein was monitored using Western blotting analyses. HEK293 cells expressing *KCNH2*-*G572S* were lysed, and the total proteins (TP) were extracted. Moreover, the total protein membrane (PM) was purified using a plasma membrane protein extraction kit according to the manufacturer's instructions (Biovision, Inc. USA). TP or PM (150 μg) per sample was separated by 10% SDS-PAGE and blotted onto nitrocellulose membrane (Stratagene, La Jolla, CA). Subsequently, *hERG* protein was detected using primary antibodies against Actin (Affinity Reagents), the specific polyclonal rabbit anti-*hERG* antibody (Santa Cruz Biotechnology, CA) and goat anti-rabbit Alexa Fluor 700 (Molecular Probes, Eugene, OR, dilution 1:2000). Densitometry and the Scion Image Software (Scion, Frederick, MD) were used to quantify the band densities. All data were normalized against Actin (*n* = 3).

### Confocal microscopy examination

HEK293 cells were transfected with different *hERG* plasmids *(pcDNA3-WT-hERG, pcDNA3-G572S-hERG* and *pcDNA3-KCNE2*). Forty eight hours later, HEK 293 cells were fixed in 4% paraformaldehyde, treated with 0.1% Triton X-100, and blocked with 3% bovine serum albumin (BSA) at room temperature. These cells were then stained with rabbit polyclonal *anti-hERG* (1:50 dilution, Alomone, Israel) and mouse monoclonal *anti-KCNE2* (1:100 dilution, Abcam, MA, USA) at 4°C overnight, followed by incubation with Alexa Fluor 488 goat anti-rabbit IgG and Alexa Fluor 555 goat anti-mouse IgG at 37°C for 2 h. Stained cells were examined under a confocal microscope (FV10i, Olympus, Japan) for subcellular location of *hERG* protein.

### The assay system for action potential

To investigate the effect of *G572S-hERG* mutation on action potential duration, we built a cell system in which sodium (Nav1.5) channels, inward rectifier potassium channel (Kir2.1) and *hERG* potassium channels were co-expressed according to Fujii reported (Fujii et al., 2012). HEK293 cells with stable expression of *SCN5A cDNA* (Nav1.5) were transfected with 2.0 μg *hERG cDNA* (Kv11.1) and 2.0 μg *KCNJ2 cDNA* (Kir2.1) according to Lipofectamine method (Life Technologies, Gaithersrburg, MD, USA) suggested by the manufacturer. *CD8 cDNA* was co-transfected to be used as a reporter gene (EBo-pCD vector, American Type Culture Collection). *CD8*-positive cells identified using Dynabeads (Dynal, M-450 CD8) were patch-clamped 48–72 h after transfection.

### Data analysis

Data are expressed as means ± S.E. Voltage protocols and data analysis were done with pCLAMP 10.2 (Axon Instruments) and Origin 6.1 (Microcal Software). Student's *t*-test was used for statistical analysis between the experimental groups (two-tailed). For multiple group comparisons, statistical significance was determined by ANOVA. With a ANOVA followed by a Student-Newman-Keuls (S-N-K) *post hoc* test, significance between any two groups was evaluated. *P*-values lower than 0.05 were considered statistically significant. The mid-point of activation was obtained using a Boltzmann function where G = [1 + exp(*V*_1/2_-*V*) /*k*]^−1^, where *V*_1/2_ and *k* are the mid-point and the slope factor, respectively, and G = I/(*V*-*V*rev) where *V*rev is the reversal potential and V is the membrane potential. The steady state of inactivation was also fitted using the Boltzmann equation. With a two-exponential function, the time constants of inactivation were assessed. All data were fitted using a nonlinear least-squares minimization method.

## Results

### Effect of *KCNE2* on *KCNH2*-*G572S* currents

**Figure 1:**
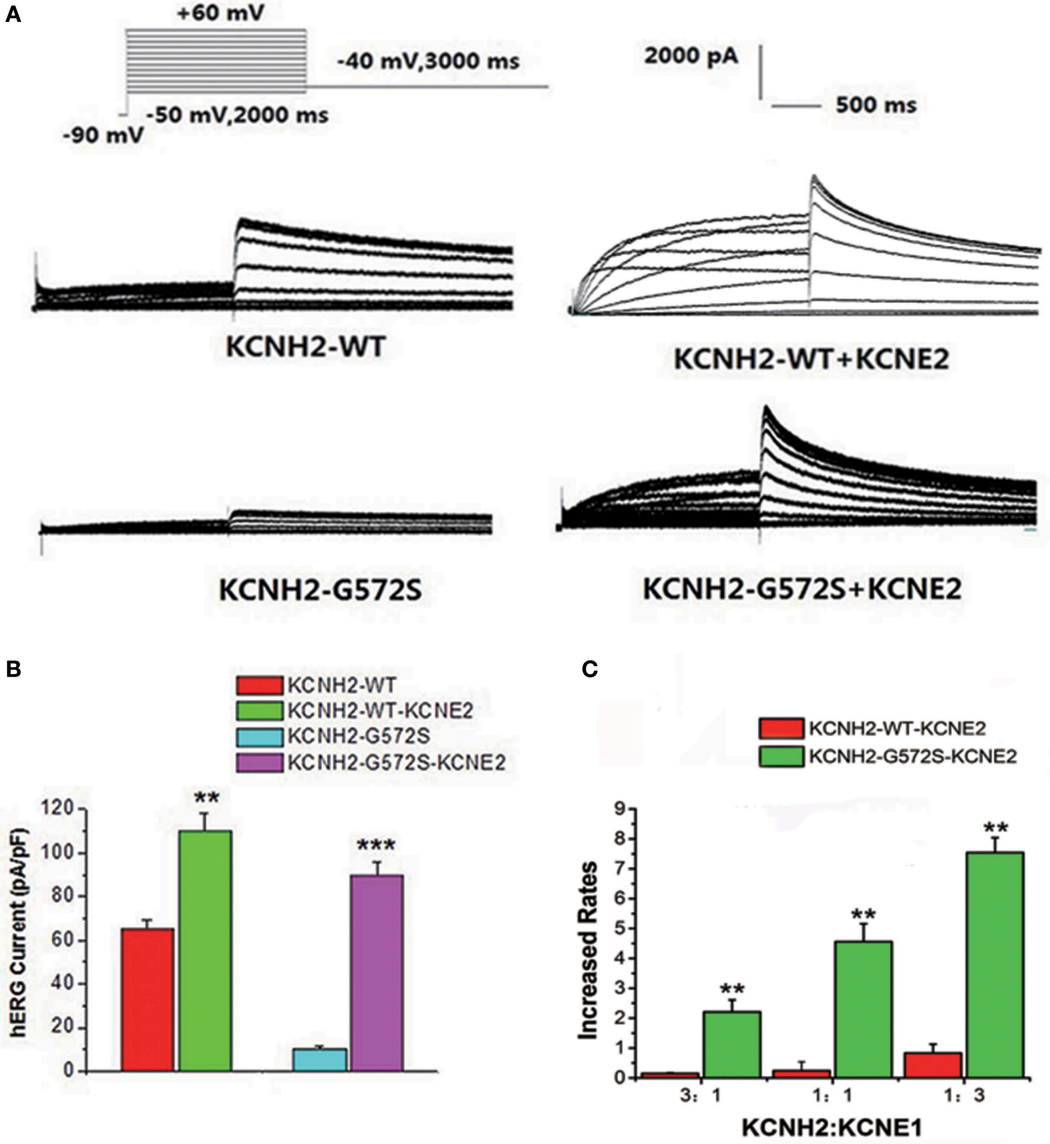
**Effect of *KCNE2* on *KCNH2*-*G572S* current**. Compared with *WT*, tail current of G572S is significantly decreased. After co-transfecting *KCNH2* and *KCNE2*, tail currents of *G572S* and WT are significantly increased **(A)**. A more significant enhancement of mutation current is found than WT by *KCNE2* at test potential of +60 mV. After co-transfecting with *KCNE2*, tail current densities of *G572S* and *WT* were both significantly increased. *KCNE2* had a more significantly enhanced effect on *G572S* mutation current densities than on *WT* **(B)**. Due to the different proportion of *KCNH2: KCNE2*, the currents are different, furthermore, current is the largest at *KCNH2: KCNE2* of 1:3. *n* = 10, ^**^P < 0.01 and ^***^P < 0.001 **(C)**.

The step and tail currents of *KCNH2* elicited upon from a holding potential of −90 mV, the membrane potential was stepped from −50 to +60 mV for 2000 ms, and then repolarized to −40 mV for 3000 ms to elicit outward tail current. The standard protocol was applied with a start-to-start interval of 15 s. First of all, to define the functional changes of the mutation *G572S*, whole-cell currents of *WT* or *G572S* mutant channels were recorded respectively, without *KCNE2*. Currents recorded from *WT* channel showed the typical *hERG* current traces: relatively slow activation and deactivation with an inwardly rectifying profile. Compared with *WT*, tail current densities of *G572S* significantly decreased. After co-transfecting with *KCNE2*, tail current densities of *G572S* and *WT* were both significantly increased. At +60 mV, tail current densities of *WT* and *G572S* before and after co-transfecting with *KCNE2* were 65.4 ± 3.6 vs. 110.4 ± 7.5 pA/pF of *WT* and 10.2 ± 1.2 vs. 89.4 ± 6.4pA/pF of *G572S* (*n* = 10, *P* < 0.01). We found that *KCNE2* had a more significantly enhanced effect on *G572S* mutation current than on *WT* (Figures 1A,B). To test the effects of *KCNE2* quantity, we used the different ratio of 3:1, 1:1, and 1:3 of *KCNH2*:*KCNE2* in the experiment. The results showed the current was largest at *KCNH2-G572S*:*KCNE2* of 1:3 (Figure 1C).

### Effect of *KCNE2* on current-voltage relationship curve of *KCNH2*-*G572S* currents

**Figure 2:**
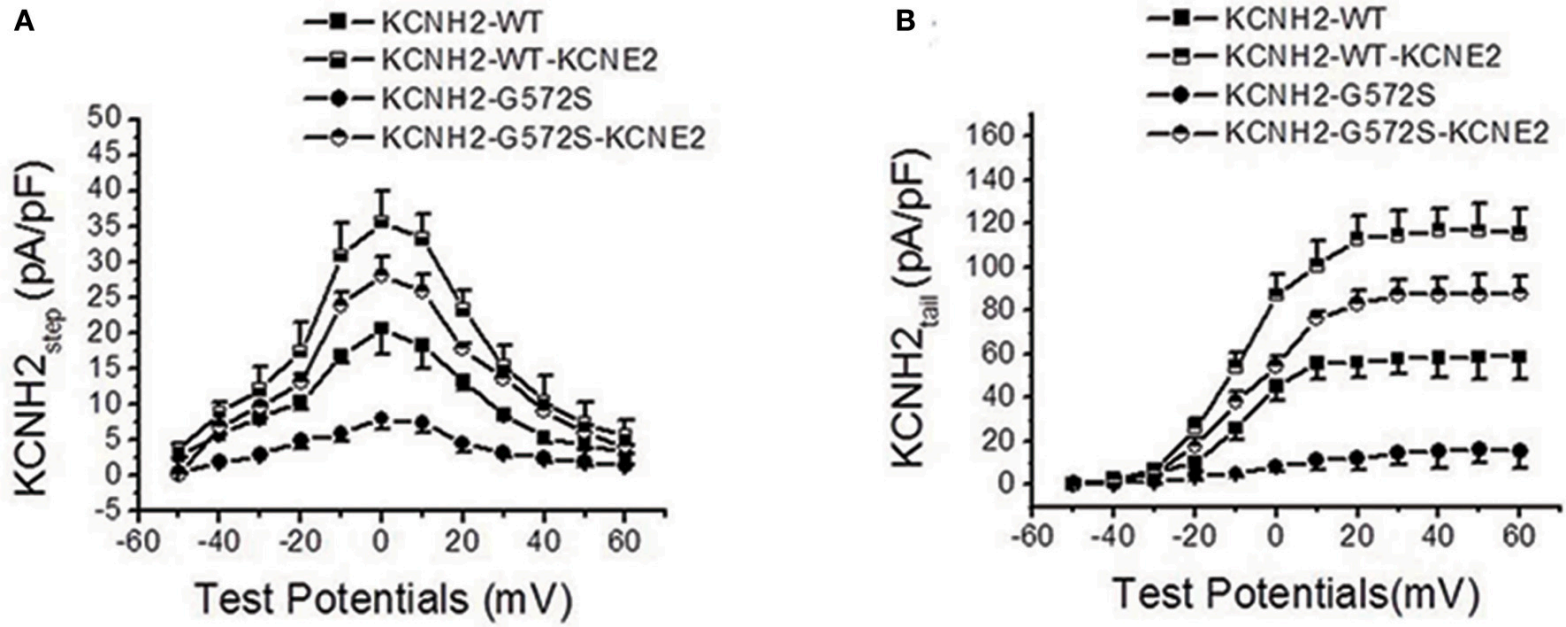
**Effect of *KCNE2* on current-voltage relationship curve of *KCNH2*-*G572S* current**. I-V relationship curve for step currents of *G572S* is lower than that of *WT* between −10 and +20 mV of test potentials and *G572S* and *WT* currents are significantly enhanced by *KCNE2* **(A)**. I-V relationship curve for tail current of *G572S* is lower than that of *WT* over +10 mV of test potentials and increase of *G572*S currents (*n* = 10, *P* < 0.001) by *KCNE2* is more significant than *WT* (*n* = 10, *P* < 0.01) **(B)**.

As shown in Figure 2A, I-V relationship curve for step currents of *G572S* was significantly lower than that of *WT* between −10 and +20 mV, indicating there was a more obvious reduction in *G572S* amplitudes compared to in *WT*. When co-expressing *KCNE2, G572S* and *WT* currents were both significantly enhanced. As shown in Figure 2B, I-V relationship curve for tail current of *G572S* was lower than that of *WT* over +10 mV of test potentials and enhancement of *G572S* currents by *KCNE2* was more prominent than that of *WT* (*n* = 10, *p* < 0.01).

### Effect of *KCNE2* on gating kinetics of *KCNH2*-*G572S* currents

**Figure 3:**
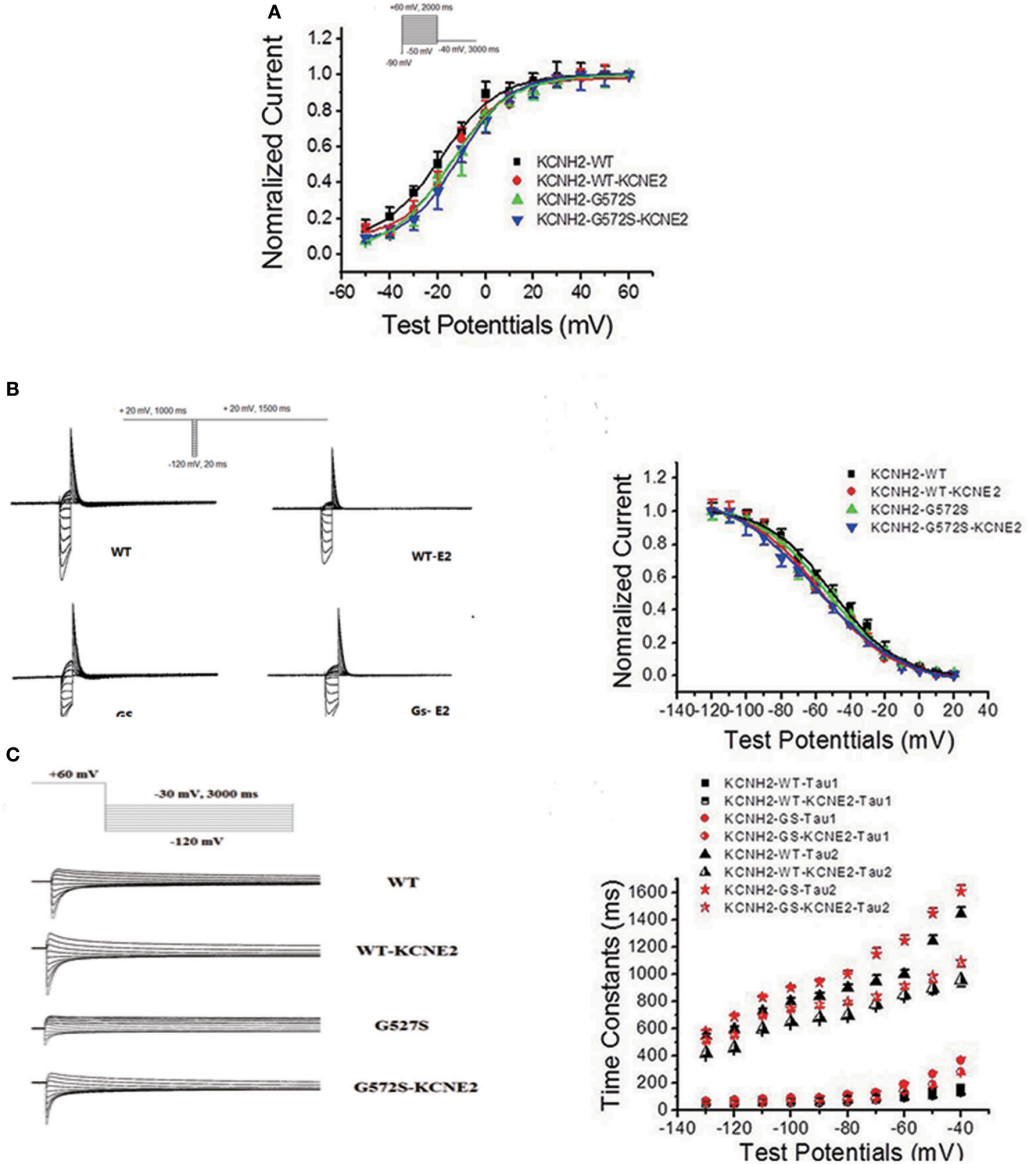
**Effect of *KCNE2* on gating kinetics of *KCNH2*-*G572S* current**. Tail current data were normalized to the maximum current value and fitted with Boltzmann function. Either V_1/2_ or k of steady-state activation and inactivation of *G572S* mutant is similar to that of *WT* and the parameters steady-state activation and inactivation do not change after co-transfecting *KCNE2* and *KCNH2* **(A,B)**. Fast time constants of deactivation of *G572S* and *WT* are similar either with or without *KCNE2*. Slow time constants of deactivation of *G572S* and *WT* with co-expressed *KCNE2*are shorter at range of test potentials **(C)**.

The steady-state activation was assessed using standard tail current analysis. Cells were depolarized to potentials in the range −60 to +30 mV (4 s) and tail current was recorded at −120 mV. The steady-state inactivation currents for *G572S* and *WT* were measured with the following protocol: Channels were inactivated under double-pulse model with condition pulse depolarization +20 mV (1 s) and following short test pulses to potentials ranging from −120 to +20 mV (15 ms, 10 mV-increments) that was applied to recover the channels from inactivation. Returning to the holding potential of +20 mV evoked large outward inactivating currents. Tail current data were normalized to the maximum current value and fitted with Boltzmann function. Either V_1/2_ or k of steady-state activation and inactivation of *G572S* mutant were similar to that of *WT*, and the parameters of steady-state activation and inactivation do not change after co-transfecting *KCNE2* and *KCNH2*. Fast time constants of deactivation of *G572S* and *WT* were similar either with or without *KCNE2*. Slow time constants of deactivation of *G572S* and *WT* with co-expressed *KCNE2* were shorter at range of test potentials (Figure 3).

### Effect of *KCNE2* on protein expression of *KCNH2*-*G572S* channel

**Figure 4:**
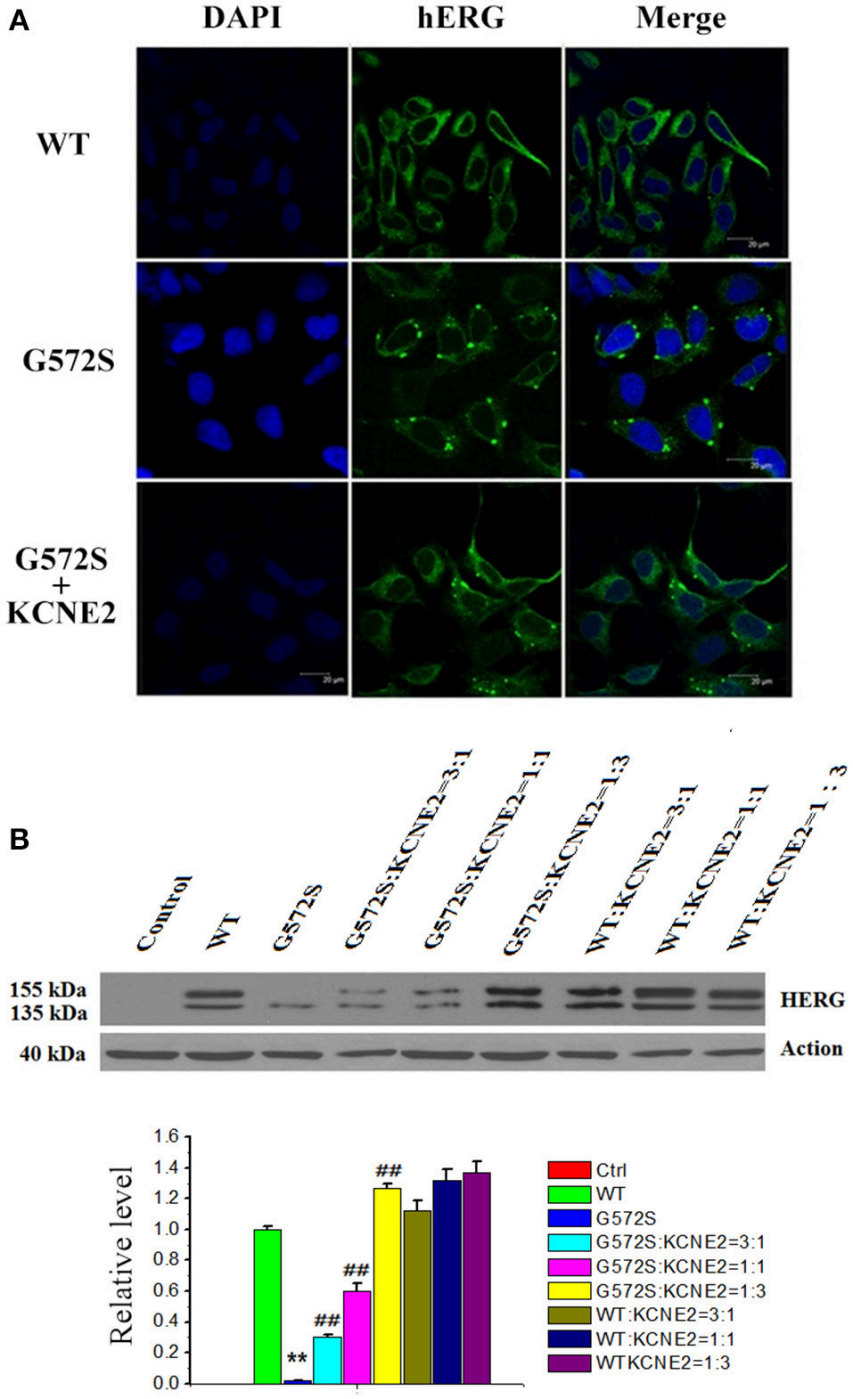
**Rescue effect of *KCNE2* on protein expression of *KCNH2*-*G572S* channel**. Confocal images were taken from the HEK293 cells expressing *WT, G572S*, and *G572S-KCNE2*, respectively. The immunofluorescence images showed the localization of *hERG* protein on cell membrane. The *hERG*-*GFP* labeling on the cell membrane was shown in green, while the chromatin-DAPI was shown in blue **(A)**. Western blot analysis of the expression of *KCNH2-G572S* and *KCNH2*-*WT* channel protein was performed in transfected HEK293 cells. *G572S* channel mature protein fraction in the region of the cell membrane (155 kDa) is markedly decreased than that of *WT*, but when co-transfecting *KCNE2*, the membrane protein (155 kDa) expression substantially increased. Due to the different proportion of *KCNH2*: *KCNE2*, the membrane protein (155 kDa) expression are different, furthermore, the expression is the largest at *KCNH2*: *KCNE2* of 1:3. *n* = 10, ^**^P < 0.01, vs. *WT* group; ^##^*P* < 0.01, vs. *G572S* group (**B**).

To further investigate the regulation of *KCNE2* on *G572S* mutation, subcellular localization of mutant proteins in the cell surface was examined. Confocal images were taken from the HEK293 cells expressing *WT, G572S*, and *G572S-KCNE2*, respectively. The images showed the localization of *hERG* protein on cell membrane. Cells expressing *WT* had a marked surface membrane localization of *hERG*; whereas the cells expressing *G572S* apparently had a reduced fluorescent intensity on membrane surface. When co-transfecting HEK293 cells with *G572S* and *KCHE2*, the fluorescent intensity on membrane surface increased (Figure 4A).

*WT* channel protein on western blot was present in both immature core-glycosylated form of 135 kDa that was localized to the ER and mature complexly glycosylated form of 155 kDa that was inserted into the cell membrane (Zhou et al., 1998; Ficker et al., 2003). To investigate the expression of *hERG* protein of *G572S* mutation, western blotting analyses of the different fractions were performed. Figure 4B showed that in the case of *WT*, co-transfecting with *KCNE2* increased the expression of *hERG* protein on the plasma membrane (155 kDa) without affecting the total protein content in whole cell homogenates. Compared with *WT, G572S* channel mature protein fraction in the region of the cell membrane (155 kDa) was markedly decreased while non-mature protein (135 kDa) had no change. Co-expressing *G572S* and *KCNE2* could cause a substantial up-regulated membrane protein (155 kDa) expression. Furthermore, expression of membrane protein accumulated markedly with increasing ratio of *KCNH2*:*KCNE2*. This result showed there was more membrane protein (155 kDa) expression at *KCNH2-G572S*:*KCNE2* of 1:3 in current study. Protein expressing both of *KCNH2-WT:KCNE2* of 3:1and *KCNH2-WT:KCNE2* of 1:1 were also increased, however, the increase rate was smaller than *KCNH2-WT:KCNE2* of 1:3. The result showed that *KCNE2* maybe had rescue effect on trafficking dysfunction of *G572S*. This experiment was repeated three times (*P* < 0.01) (Figure 4B).

### Rescue effects of *KCNE2* on *G572S* mutation under the condition of low temperature

**Figure 5:**
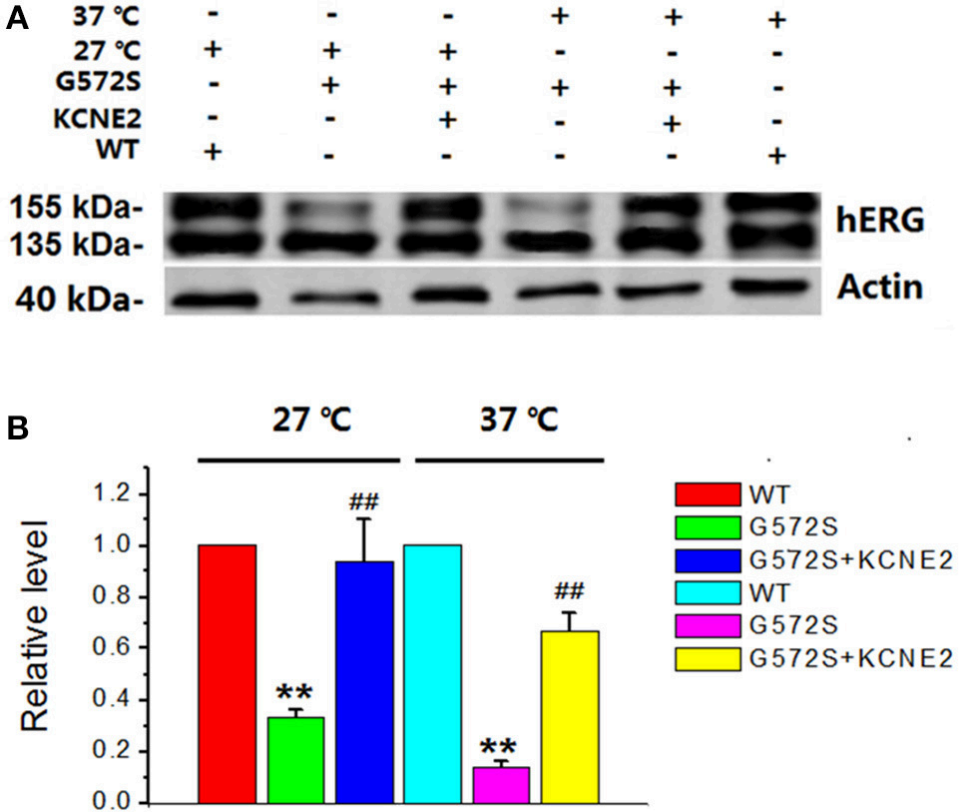
**Rescue effects of *KCNE2* on *G572S* mutation at lower temperature**. Representative western blot analysis of the *hERG* protein expression was performed at 37°C and 27°C with/without *KCNE2*, respectively **(A)**. The matured protein of *G572S* was increased at 27°C compared with at 37°C, which was inferred by the appearance of the 155-kDa protein in western blot analysis. Furthermore, co-transfecting *G572S* with *KCNE2* led to another substantial increase of membrane protein expression both at 37° and 27°C. (*n* = 3) ^**^P < 0.01, vs. *WT* group; ^##^*P* < 0.01, vs. *G572S* group **(B)**.

It has been reported that there is temperature-dependent rescuing effect in some trafficking-deficient mutations of *hERG* protein, such as *R752W, G601S*, and *N470D*. Low temperature culturing of cells has been reported to increase the surface expression of many *hERG* mutants. To examine whether this temperature-dependent rescuing effect worked for *G572S* mutant, representative western blot analysis of *hERG* protein expression was performed at 37°C and 27°C, respectively. It was obtained that matured protein (155 kDa) of *G572S* mutant were 31.6% at 27°C and 17.2% at 37°C. This result showed that exposure of cells at 27°C restored the matured protein (155 kDa) expression of *G572S*, compared with at 37°C. With co-expression of *KCNE2*, matured protein (155 kDa) of *G572S* mutant were increased to 92.5% at 27°C and 68.4% at 37°C, respectively. These results suggested that lower temperature could be the favorable circumstance for the rescue function of *KCNE2* (Figure 5).

### Building of “the action potential (AP)” by co-transfecting *SCN5A, KCNJ2*, and *KCNH2* into HEK293 cells

**Figure 6:**
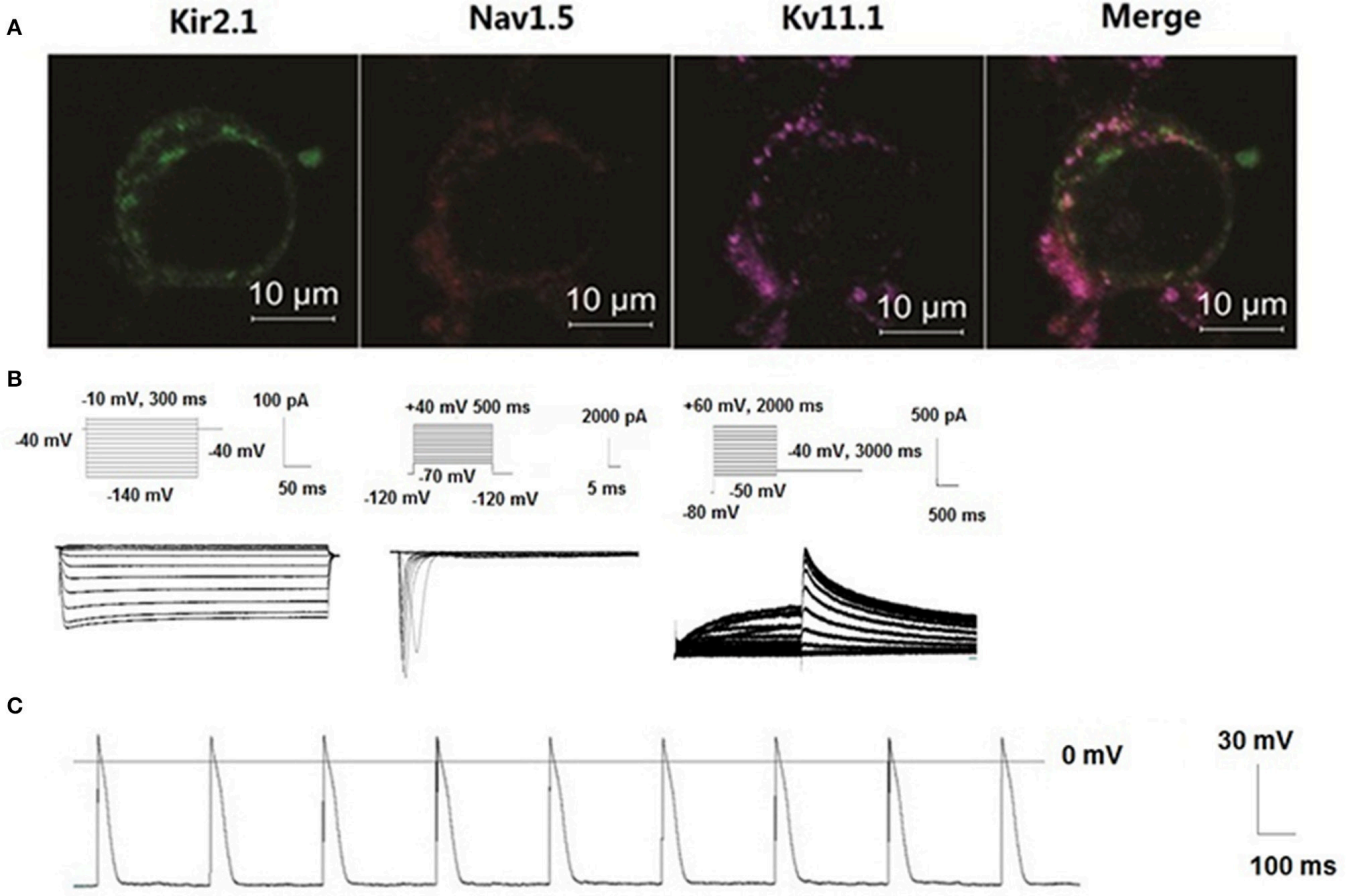
**New building of the action potential by co-transfecting Nav1.5, Kir2.1, and *KCNH2* into HEK293 cells**. Distribution of three channels was detected by confocal microscopy. Images demonstrated that Nav1.5, Kir2.1, and Kv11.1 proteins were stable co-expressed on the HEK293 cell membrane **(A)**. Schematic representation of the voltage-clamp protocol and the representative Nav1.5, Kir2.1, and *KCNH2* current traces **(B)**. Action potentials were structured by Nav1.5, Kir2.1, and Kv11.1 channel porteins in the HEK293 cells **(C)**.

HEK 293 cell line was utilized as a proof-of-concept unexcitable somatic cell source based on its low levels of endogenous membrane currents, uniform shape and growth, and extensive use as a heterologous expression system for studies of ion channel function (Thomas and Smart, 2005). We chose three basic components of action potential. *KCNJ2* (Kir2.1,) channel maintained the resting membrane potential, *SCN5A* (Nav1.5) channel mainly contributed 0 phase depolarzion of AP, and *KCNH2* (Kv11.1) had critical role in repolarization of AP. To verify subcellular localization of Nav1.5, Kir2.1, and Kv11.1 proteins, distribution of three channels was detected by confocal microscopy. Images demonstrated that Nav1.5, Kir2.1, and Kv11.1 proteins were stable co-expressed on the HEK293 cell membrane (Figure 6A). To assess whether these proteins expressing on cell membrane were functional, we recorded I_Na_, I_K1_, and I_Kr_ currents in voltage clamp mode using whole-cell patch-clamp technique respectively (Figure 6B). Three currents were recorded, which indicated co-transfection or co-expression was successful. Subsequently, we applied current injection ranged from 900 to 1500 pA for 5 ms to stimulus HEK293 cell. AP was found on the oscilloscope with a shorter AP duration with 9.2 ± 0.2 ms of 50% repolarization of APD (APD_50_) and 49.5 ± 2.7 ms of 90% repolarization of APD (APD_90_). Action potential amplitudes (APA) were 108.5 ± 3.4 mV and resting membrane potentials (RMP) were −70.1 ± 1.2 mV (Figure 6C).

### Rescue effect of *KCNE2* on action potential composed of *KCNH2-G572S*

**Figure 7:**
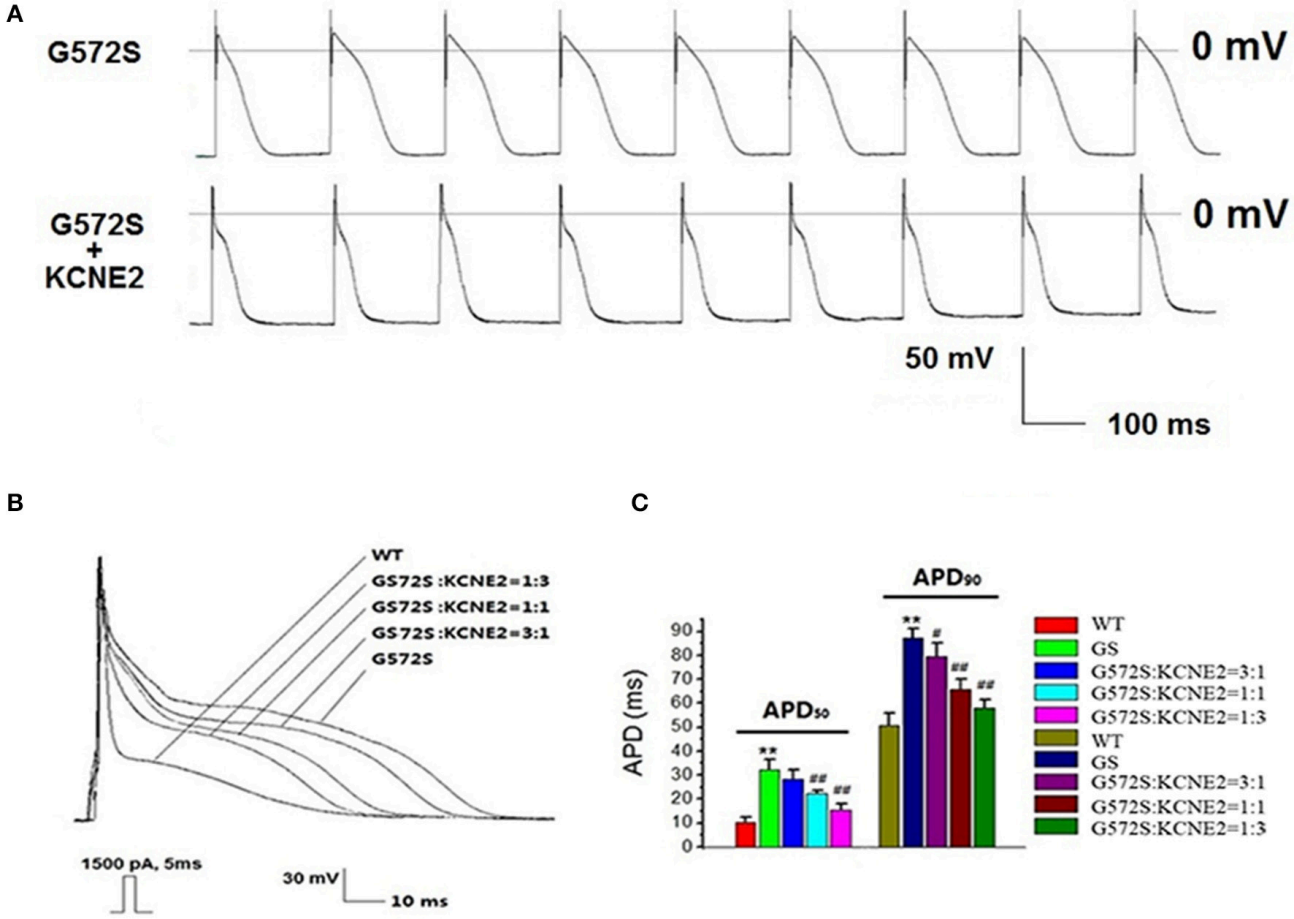
**Rescue effect of *KCNE2* on action potential composed of *KCNH2*-*G572S***. New building action potential was recorded by using clamp-patch technique **(A)**. It was found that prolongation of APD_50_ and APD_90_ of *G572S* mutant were both significantly shortened by *KCNE2*. Due to the different proportion of *KCNH2*: *KCNE2*, this rescue effect by *KCNE2* were different, furthermore, the most significantly rescue effect was at *KCNH2*: *KCNE2* of 1:3. (*n* = 10), ^**^P < 0.01, vs. *WT* group; ^#^*P* < 0.05, vs. *G572S* group; ^##^*P* < 0.01, vs. *G572S* group **(B,C)**.

To demonstrate the contribution of *KCNE2* on shortening the APD prolonged by *G572S*, we established Kir2.1+Nav1.5+Kv11.1*-G572S* HEK293 cell line, and AP was recorded with/without co-expressing *KCNE2*. Figure 7A showed the two representative AP traces and visually, *KCNE2* could shorten the APD which was prolonged by *G572S*. APD_90_ of *G572S* and *WT* were 85.7 ± 4.5 ms and 49.5 ± 2.7 ms respectively. APD_90_ of *G572S* after co-transfecting with *KCNE2* were shortened to 56.2 ± 3.3 ms (*n* = 10, *P* < 0.01). This suggested that *KCNE2* could rescue APD prolonged by *G572S*. Furthermore, different ratio of 3:1, 1:1, and 1:3 of *KCNH2*:*KCNE2* was used in the experiment. The most significantly rescue effect was at *KCNH2*: *KCNE2* of 1:3 which was in accord with the effect of *KCNE2* on *G572S* mutation current. (*n* = 10, *P* < 0.01, Figures 7B,C).

## Discussion

In this experiment, we first found *KCNE2* could significantly increase the tail current densities of *KCNH2*-*G572S* mutation. Previous experiments have shown that *KCNH2*-*G572S* mutation led to loss of channel function, with a significantly lower current and a markedly prolongation of action potential duration and QT interval in electrocardiogram (Splawski et al., 2000; Napolitano et al., 2005; Tester et al., 2005). Our results suggest that *KCNE2* restores the channel function, increases both *WT* and *G572S* current and shortens the APD prolonged by *G572S*.

Protein processing and trafficking may play an important and regulated role in controlling channel characteristics (Geva and Schuldiner, 2014). To our knowledge, there are two main reasons why the loss of function happen following channel gene mutant (Sanguinetti et al., 1996; Thomas, 2003): (1). Change of the gating dynamics causes a decline current; (2). The trafficking defect of channel protein to the cell membrane gives rise to down-regulation of the membrane channel proteins and reduces current. *hERG* channels are translated and initially glycosylated at the endoplasmic reticulum (ER, molecular weight of *hERG* protein is 135 kDa) (Petrecca et al., 1999). After folding properly with the assistance of chaperones, *hERG* channels are trafficking to the Golgi for secondary glycosylation (molecular weight of *hERG* protein is 155 kDa) and then further trafficking to the membrane (Gong et al., 2002; Smith et al., 2011). In this study, our results showed that the cells expressing *G572S* apparently had a reduced fluorescent intensity and maturation membrane protein (155 kDa) expression on membrane surface. These rusults suggested that *G572S* maybe led to channel protein migration defects. We have focused on the physical protein-protein association and trafficking of *KCNE2* and *hERG*. After co-transfection with *KCNE2*, membrane protein expression of *G572S* mutation and *hERG* currents densities are increased significantly. The small proteins encoded by *KCNE2* have been proposed as accessory subunits for the *hERG* channel (Abbott et al., 1999). So, it is supported the concept that assembly of *KCNE* proteins with K^+^ channels happens early in the biosynthetic process, probably in the ER (Shi et al., 1996; Krumerman et al., 2004; McKeown et al., 2008). *KCNE2* can promote the Kv11.1 channel protein to traffic the cell surface and expression of membrane protein (Um and McDonald, 2007). This may partly explain the rescue mechanism of *KCNE2* on *G572S* mutations in this study.

Mutations in different locations of the *hERG* potassium-channel gene are associated with different levels of risk for arrhythmic cardiac events in LQT2, with the greatest risk related to mutations in the critical pore region of the channel (Moss et al., 2002). *G572S* is a *hERG* pore domain mutation which locates in the short linker between the fifth transmembrane domain and the 5-P α-helix of the pore domain. The placement of the inactivation helix (between the S5- and pore helix) is based on functional studies. Anderson et al. speculated that the location of amino acid substitutions in putative Kv11.1-helical or-sheet domains appears to correlate with the trafficking-deficient and mutations disrupted Kv11.1 channel biogenesis (Anderson et al., 2006). Based on this speculation, we hypothesize that *KCNE2* propably correct the change of the protein space structure caused by *G572S*, and this correction could stabilize the channel protein in configurations that facilitate proper trafficking.

As we have known, at lower temperatures, an increased ER retention time might make an improved channel folding. Additionally, a reduced incubation temperature could reverse the mutant's effects on *hERG* currents according to inhibition of proteasomal degradation and therefore increase the surface expression of these *hERG* mutants (Thomas et al., 2003). At physiological temperatures mutant channels cannot pass quality control processes of the ER and do not reach the plasma membrane. At reduced temperatures, protein folding might be altered and ultimately permitted exit from the ER (Ficker et al., 2000). It has been reported that low temperature could correct *hERG* defective protein trafficking, thus increase the surface expression of some LQT2-causing mutants, such as *G601S, K28E, N407D*, and *A422T* (Zhou et al., 1999; Gong et al., 2006; Chen et al., 2007; Guo et al., 2012). *G572S* has a dominant negative trafficking defect and a severe phenotype (Zhao et al., 2009). In our research, matured protein expression of *G572S* was found to be restored by lower incubation temperature, which is similar to those mutations with trafficking defect mentioned above. This result supported *G572S* was likely a temperature-sensitive mutation localized to the transmembrane domain. Based on our observation, *KCNE2* induced a more effectively up-regulation of matured protein (155 kDa) expression of *G572S* at lower temperature, which has not been reported before. However, the molecular mechanism responsible for this phenomenon remains to be analyzed in the future.

In the study about mechanism of ion channel diseases, it is indispensable to evaluate the effect of mutation channels on the action potential. However, subculture of the myocardial cell isolation is difficult. Other difficulties lie in cellular toxicity of transfection reagents and low transfection efficiency. Besides, low differentiation rate of induced pluripotent stem cells (iPS) limits to its application (Müller et al., 2013). Although computer simulation can better reflect the effects of mutations channel of action potential (ten Tusscher et al., 2004), it is unable to objectively reflect the real situation. It was reported that the HEK 293 cells genetically engineered to stably express both *SCN5A, KCNJ2*, much like primary excitable cells, could develop membrane excitability. wherein on reaching an excitation threshold by current injection, they reproducibly fired an “all-or-none” AP (Kirkton and Bursac, 2011). We chose resting potential current of I_K1_, depolarization current of I_Na_ and major repolarization current of I_Kr_ to build an action potential and then a typical sample of myocardial cell-like action potential was recorded. However, it seems to be different from the classic AP. The possible reason might be a lack of calcium channel which contributes to the repolaration plateau of action potential.

By using the above system, we found *hERG-G572S* was a loss of function mutation which could lead a reduction of *hERG* current densities and could prolong APD of HEK 293 cell line established in our research. However, with *KCNE2* co-expression, *hERG* current density increased and the APD was significantly shortened. This assay system which was used to evaluate function of *hERG* channel genetic mutations in our research is a useful tool because of its simplicity and high cost performance.

The findings in this study described electrophysiological characteristics of the LQT2 syndrome mutation *KCNH2-G572S* and regulation by accessory protein *KCNE2*. The study provided a clue about LQT2 and relative rescue mechanism. It might prompt a certain treatment of *KCNE2* on LQT2.

## Author contributions

All authors contributed to this work significantly. LL, contributed to study design, performed site-directed mutagenesis, transfection, and patch clamp experiments independently, interpreted results of experiments, drafted manuscript, prepared figures. JT, performed western blotting experiments, analyzed data, interpreted results of experiments, drafted manuscript. CL, performed confocal microscopy examination, interpreted results of experiments, revised the manuscript. XC, YF, BX, CZ, participated in interpreting results of experiments, edited the manuscript. YS, YUZ, YIZ, revised the manuscript. YL, corresponding author, contributed to conception and design of research, analyzed, and interpreted data of experiments, prepared figures, drafted and revised the manuscript, and accounted for all aspects of the work. All authors read and approved the final version of the manuscript.

### Conflict of interest statement

The authors declare that the research was conducted in the absence of any commercial or financial relationships that could be construed as a potential conflict of interest.

## References

[B1] Abbott G. W. Sesti F. Splawski I. Buck M. E. Lehmann M. H. Timothy K. W.. (1999). MiRP1 forms IKr potassium channels with HERG and is associated with cardiac arrhythmia. Cell 97, 175–187. 10219239 10.1016/s0092-8674(00)80728-x

[B2] Anderson C. L. Delisle B. P. Anson B. D. Kilby J. A. Will M. L. Tester D. J.. (2006). Most LQT2 mutations reduce Kv11.1 (hERG) current by a class 2 (trafficking-deficient) mechanism. Circulation 113, 365–373. 10.1161/CIRCULATIONAHA.105.570200 16432067

[B3] Chen M. X. Sandow S. L. Doceul V. Chen Y. H. Harper H. Hamilton B.. (2007). Improved functional expression of recombinant human ether-a-go-go (hERG) K+ channels by cultivation at reduced temperature. BMC Biotechnol 7:93. 10.1186/1472-6750-7-93 18096051 PMC2241608

[B4] Dupuis D. S. Klaerke D. A. Olesen S. P. (2005). Effect of β-adrenoceptor blockers on human ether-a-go-go-related gene (HERG) potassium channels. Basic Clin. Pharmacol. Toxicol. 96, 123–130. 10.1111/j.1742-7843.2005.pto960206.x 15679475

[B5] Eldstrom J. Fedida D. (2011). The voltage-gated channel accessory protein KCNE2: multiple ion channel partners, multiple ways to long QT syndrome. Expert Rev. Mol. Med. 13, e38. 10.1017/S1462399411002092 22166675

[B6] Ficker E. Dennis A. T. Wang L. Brown A. M. (2003). Role of the cytosolic chaperones Hsp70 and Hsp90 in maturation of the cardiac potassium channel HERG. Circ. Res. 92, e87–e100. 10.1161/01.RES.0000079028.31393.15 12775586

[B7] Ficker E. Thomas D. Viswanathan P. C. Dennis A. T. Priori S. G. Napolitano C.. (2000). Novel characteristics of a misprocessed mutant HERG channel linked to hereditary long QT syndrome. Am. J. Physiol. Heart Circ. Physiol. 279, H1748–H1756. 11009462 10.1152/ajpheart.2000.279.4.H1748

[B8] Fujii M. Hayashi K. Ohya S. Yamamura H. Imaizumi Y. (2013). New screening system for selective blockers of voltage-gated K(+) channels using recombinant cell lines dying upon single action potential. J. Pharmacol. Sci. 123, 147–158. 24096832 10.1254/jphs.13063fp

[B9] Fujii M. Ohya S. Yamamura H. Imaizumi Y. (2012). Development of recombinant cell line co-expressing mutated Nav1.5, Kir2.1, and hERG for the safety assay of drug candidates. J. Biomol. Screen. 17, 773–784. 10.1177/1087057112442102 22498908

[B10] Geva Y. Schuldiner M. (2014). The back and forth of cargo exit from the endoplasmic reticulum. Curr. Biol. 24, R130–R136. 10.1016/j.cub.2013.12.008 24502791

[B11] Gong Q. Anderson C. L. January C. T. Zhou Z. (2002). Role of glycosylation in cell surface expression and stability of HERG potassium channels. Am. J. Physiol. Heart Circ. Physiol. 283, H77–H84. 10.1152/ajpheart.00008.2002 12063277

[B12] Gong Q. Jones M. A. Zhou Z. (2006). Mechanisms of pharmacological rescue of trafficking-defective hERG mutant channels in human long QT syndrome. J. Biol. Chem. 281, 4069–4074. 10.1074/jbc.M511765200 16361248 PMC1624912

[B13] Guo J. Zhang X. Hu Z. Zhuang Z. Zhu Z. Chen Z. (2012). A422T mutation in HERG potassium channel retained in ER is rescurable by pharmacologic or molecular chaperones. Biochem. Biophys. Res. Commun. 422, 305–310. 10.1016/j.bbrc.2012.04.153 22580281

[B14] Kirkton R. D. Bursac N. (2011). Engineering biosynthetic excitable tissues from unexcitable cells for electrophysiological and cell therapy studies. Nat. Commun. 2, 300. 10.1038/ncomms1302 21556054 PMC3388000

[B15] Krumerman A. Gao X. Bian J. S. Melman Y. F. Kagan A. McDonald T. V. (2004). An LQT mutant minK alters KvLQT1 trafficking. Am. J. Physiol. Cell. Physiol. 286, C1453–C1463. 10.1152/ajpcell.00275.2003 14761891

[B16] Mazhari R. Greenstein J. L. Winslow R. L. Marbán E. Nuss H. B. (2001). Molecular interactions between two long-QT syndrome gene products, HERG and KCNE2, rationalized by *in vitro* and *in silico* analysis. Circ. Res. 89, 33–38. 11440975 10.1161/hh1301.093633

[B17] McKeown L. Swanton L. Robinson P. Jones O. T. (2008). Surface expression and distribution of voltage-gated potassium channels in neurons (Review). Mol. Membr. Biol. 25, 332–343. 10.1080/09687680801992470 18446619

[B18] Moss A. J. Zareba W. Kaufman E. S. Gartman E. Peterson D. R. Benhorin J.. (2002). Increased risk of arrhythmic events in long-QT syndrome with mutations in the pore region of the human ether-a-go-go-related gene potassium channel. Circulation 105, 794–799. 11854117 10.1161/hc0702.105124

[B19] Müller M. Seufferlein T. Illing A. Homann J. (2013). Modelling human channelopathies using induced pluripotent stem cells: a comprehensive review. Stem Cells Int. 2013:496501. 10.1155/2013/496501 23766769 PMC3666272

[B20] Napolitano C. Priori S. G. Schwartz P. J. Bloise R. Ronchetti E. Nastoli J.. (2005). Genetic testing in the long QT syndrome: development and validation of an efficient approach to genotyping in clinical practice. JAMA 294, 2975–2980. 10.1001/jama.294.23.2975 16414944

[B21] Petrecca K. Atanasiu R. Akhavan A. Shrier A. (1999). N-linked glycosylation sites determine HERG channel surface membrane expression. J. Physiol. 515(Pt 1), 41–48. 10.1111/j.1469-7793.1999.041ad.x 9925876 PMC2269130

[B22] Saenen J. B. Vrints C. J. (2008). Molecular aspects of the congenital and acquired Long QT Syndrome: clinical implications. J. Mol. Cell Cardiol. 44, 633–646. 10.1016/j.yjmcc.2008.01.006 18336833

[B23] Sanguinetti M. C. Curran M. E. Spector P. S. Keating M. T. (1996). Spectrum of HERG K+-channel dysfunction in an inherited cardiac arrhythmia. Proc. Natl. Acad. Sci. U.S.A. 93, 2208–2212. 10.1073/pnas.93.5.2208 8700910 PMC39936

[B24] Sanguinetti M. C. Jiang C. Curran M. E. Keating M. T. (1995). A mechanistic link between an inherited and an acquired cardiac arrhythmia: HERG encodes the IKr potassium channel. Cell 81, 299–307. 7736582 10.1016/0092-8674(95)90340-2

[B25] Schwartz P. J. Crotti L. Insolia R. (2012). Long-QT syndrome: from genetics to management. Circ. Arrhythm. Electrophysiol. 5, 868–877. 10.1161/CIRCEP.111.962019 22895603 PMC3461497

[B26] Schweigmann U. Biliczki P. Ramirez R. J. Marschall C. Takac I. Brandes R. P.. (2014). Elevated heart rate triggers action potential alternans and sudden death. translational study of a homozygous KCNH2 mutation. PLoS ONE 9:e103150. 10.1371/journal.pone.0103150 25140878 PMC4139196

[B27] Shi G. Nakahira K. Hammond S. Rhodes K. J. Schechter L. E. Trimmer J. S. (1996). β subunits promote K+ channel surface expression through effects early in biosynthesis. Neuron 16, 843–852. 8608002 10.1016/s0896-6273(00)80104-x

[B28] Smith J. L. McBride C. M. Nataraj P. S. Bartos D. C. January C. T. Delisle B. P. (2011). Trafficking-deficient hERG K(+) channels linked to long QT syndrome are regulated by a microtubule-dependent quality control compartment in the ER. Am. J. Physiol. Cell Physiol. 301, C75–C85. 10.1152/ajpcell.00494.2010 21490315 PMC3129823

[B29] Sokolov S. Peters C. H. Rajamani S. Ruben P. C. (2013). Proton-dependent inhibition of the cardiac sodium channel Nav1.5 by ranolazine. Front. Pharmacol. 4:78. 10.3389/fphar.2013.00078 23801963 PMC3689222

[B30] Splawski I. Shen J. Timothy K. W. Lehmann M. H. Priori S. Robinson J. L.. (2000). Spectrum of mutations in long-QT syndrome genes. KVLQT1, HERG, SCN5A, KCNE1, and KCNE2. Circulation 102, 1178–1185. 10973849 10.1161/01.cir.102.10.1178

[B31] ten Tusscher K. H. Noble D. Noble P. J. Panfilov A. V. (2004). A model for human ventricular tissue. Am. J. Physiol. Heart Circ. Physiol. 286, H1573–H1589. 10.1152/ajpheart.00794.2003 14656705

[B32] Tester D. J. Will M. L. Haglund C. M. Ackerman M. J. (2005). Compendium of cardiac channel mutations in 541 consecutive unrelated patients referred for long QT syndrome genetic testing. Heart Rhythm 2, 507–517. 10.1016/j.hrthm.2005.01.020 15840476

[B33] Thomas D. (2003). Defective protein trafficking in hERG-associated hereditary long QT syndrome (LQT2): molecular mechanisms and restoration of intracellular protein processing. Cardiovasc. Res. 60, 235–241. 10.1016/j.cardiores.2003.08.002 14613852

[B34] Thomas D. Kiehn J. Katus H. A. Karle C. A. (2003). Defective protein trafficking in hERG-associated hereditary long QT syndrome (LQT2): molecular mechanisms and restoration of intracellular protein processing. Cardiovasc. Res. 60, 235–241. 10.1016/j.cardiores.2003.08.002 14613852

[B35] Thomas P. Smart T. G. (2005). HEK293 cell line: a vehicle for the expression of recombinant proteins. J. Pharmacol. Toxicol. Methods 51, 187–200. 10.1016/j.vascn.2004.08.014 15862464

[B36] Trudeau M. C. Warmke J. W. Ganetzky B. Robertson G. A. (1995). HERG, a human inward rectifier in the voltage-gated potassium channel family. Science 269, 92–95. 7604285 10.1126/science.7604285

[B37] Um S. Y. McDonald T. V. (2007). Differential association between HERG and KCNE1 or KCNE2. PLoS ONE 2:e933 10.1371/journal.pone.0000933 17895974 PMC1978535

[B38] Walker V. E. Atanasiu R. Lam H. Shrier A. (2007). Co-chaperone FKBP38 promotes HERG trafficking. J. Biol. Chem. 282, 23509–23516. 10.1074/jbc.M701006200 17569659

[B39] Warmke J. W. Ganetzky B. (1994). A family of potassium channel genes related to eag in Drosophila and mammals. Proc. Natl. Acad. Sci. U.S.A. 91, 3438–3442. 10.1073/pnas.91.8.3438 8159766 PMC43592

[B40] Weerapura M. Nattel S. Chartier D. Caballero R. Hébert T. E. (2002). A comparison of currents carried by HERG, with and without coexpression of MiRP1, and the native rapid delayed rectifier current. Is MiRP1 the missing link? J. Physiol. 540, 15–27. 10.1113/jphysiol.2001.013296 11927665 PMC2290231

[B41] Zhang M. Jiang M. Tseng G. N. (2001). minK-related peptide 1 associates with Kv4.2 and modulates its gating function: potential role as β subunit of cardiac transient outward channel? Circ. Res. 88, 1012–1019. 10.1161/hh1001.090839 11375270

[B42] Zhang M. Wang Y. Jiang M. Zankov D. P. Chowdhury S. Kasirajan V.. (2012). KCNE2 protein is more abundant in ventricles than in atria and can accelerate hERG protein degradation in a phosphorylation-dependent manner. Am. J. Physiol. Heart Circ. Physiol. 302, H910–H922. 10.1152/ajpheart.00691.2011 22180649 PMC3322735

[B43] Zhao J. T. Hill A. P. Varghese A. Cooper A. A. Swan H. Laitinen-Forsblom P. J.. (2009). Not all hERG pore domain mutations have a severe phenotype: G584S has an inactivation gating defect with mild phenotype compared to G572S, which has a dominant negative trafficking defect and a severe phenotype. J. Cardiovasc. Electrophysiol. 20, 923–930. 10.1111/j.1540-8167.2009.01468.x 19490267

[B44] Zhou Z. Gong Q. January C. T. (1999). Correction of defective protein trafficking of a mutant HERG potassium channel in human long QT syndrome. Pharmacological and temperature effects. J. Biol. Chem. 274, 31123–31126. 10.1074/jbc.274.44.31123 10531299

[B45] Zhou Z. Gong Q. Ye B. Fan Z. Makielski J. C. Robertson G. A.. (1998). Properties of HERG channels stably expressed in HEK 293 cells studied at physiological temperature. Biophys. J. 74, 230–241. 10.1016/S0006-3495(98)77782-3 9449325 PMC1299377

